# Phylogenetically Clustered Extinction Risks Do Not Substantially Prune the Tree of Life

**DOI:** 10.1371/journal.pone.0023528

**Published:** 2011-08-10

**Authors:** Rakesh K. Parhar, Arne Ø. Mooers

**Affiliations:** 1 Biological Sciences, Simon Fraser University, Burnaby, British Columbia, Canada; 2 IRMACS, Simon Fraser University, Burnaby, British Columbia, Canada; J. Craig Venter Institute, United States of America

## Abstract

Anthropogenic activities have increased the rate of biological extinction many-fold. Recent empirical studies suggest that projected extinction may lead to extensive loss to the Tree of Life, much more than if extinction were random. One suggested cause is that extinction risk is heritable (phylogenetically patterned), such that entire higher groups will be lost. We show here with simulation that phylogenetically clustered extinction risks are necessary but not sufficient for the extensive loss of phylogenetic diversity (PD) compared to random extinction. We simulated Yule trees and evolved extinction risks at various levels of heritability (measured using Pagel's 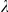). At most levels of heritability (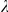 in range of 0 to 10), mean values of extinction risk (range 0.25 to 0.75), tree sizes (64 to 128 tips), tree balance and temporal heterogeneity of diversification rates (Yule and coalescent trees), extinction risks do not substantially increase the loss of PD in these trees when compared to random extinction. The maximum loss of PD (20% above random) was only associated with the combination of extremely excessive values of phylogenetic signal, high mean species' extinction probabilities, and extreme (coalescent) tree shapes. Interestingly, we also observed a decline in the rate of increase in the loss of PD at high phylogenetic clustering 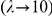 of extinction risks. Our results suggest that the interplay between various aspects of tree shape and a predisposition of higher extinction risks in species-poor clades is required to explain the substantial pruning of the Tree of Life.

## Introduction

**Figure 1:**
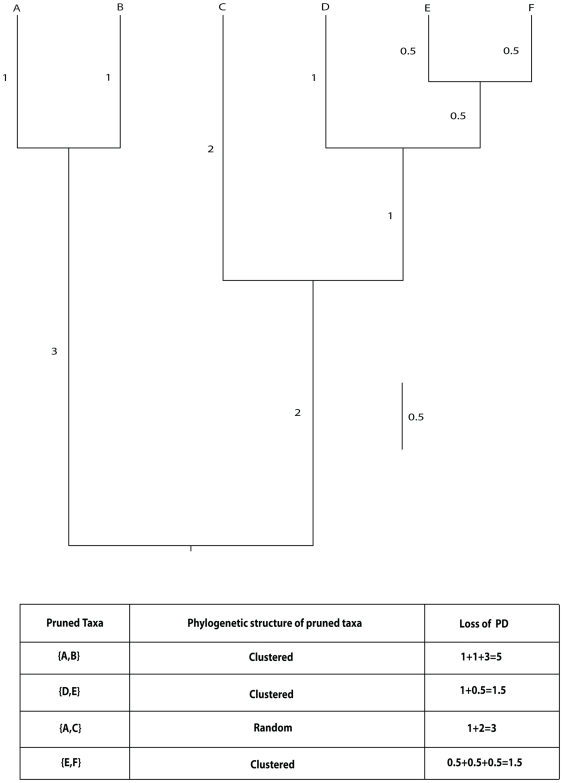
Observed PD loss on a tree. A cartoon tree of size n = 6, showing how losing different pairs of species lead to different PD loss. For this tree, total PD  = 12.5.

Phylogenetic trees estimate the evolutionary relationships among species inferred from empirical data. The edge lengths of these trees represent accrued change or temporal accounts of diversification events [1], [2]. The sum of the edge lengths has been referred to as “evolutionary history” [EH; 3]. Every time a lineage (for example, a species) goes extinct, EH is lost. This loss can be conceptualized as a pruning of the twigs and branches from the Tree of Life. However, branches on the tree that are shared by multiple species are lost only if all the subtending species go extinct (see Figure 1). A related term is phylogenetic diversity [PD; 4], a measure of the length of the subtree connecting a subset of extant species to the root of a reference phylogenetic tree. The original PD of a clade is a function of its size (the number of tips), its depth (the distance from tips to root) and its shape; for a given depth and size, the star phylogeny [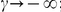 5] has maximum total PD. If one assigns a probability of extinction [p(ext)] (say, over the next 100 years) to the tips of a tree, then it is straightforward to estimate its expected future PD [E(PD); 1, 2, 6]. The difference from the original PD is the expected loss of PD.

Nee and May [3] were the first to show that there was a minimal loss of PD under homogeneous random extinction (commonly referred to as a “field of bullets” model) on a particular set of model trees. This result has been cited many times [7]–[12]. In contrast, empirical studies using real trees and estimated extinction probabilities from the IUCN red list [13] project fairly large losses of PD [7], [12], [14]–[16]. This discrepancy has been explained as being due, in part, to heritable (phylogenetically clustered) extinction risks, where related species have similar probabilities of extinction [7], [17], [18]. Indeed, phylogenetically clumped extinction can result in the loss of deeper branches in the Tree of Life [7], [11], [19]–[21]. However, Heard and Mooers [22] cautioned that heritable extinction probabilities alone might not be enough to cause large losses of PD relative to the field of bullets model. In spite of their caution 10 years ago, recent studies continue to reason that phylogenetically clustered extinction threats are responsible for the substantial loss of PD from the Tree of Life [7], [11], [19]–[21].

We return to this simple question here, and try to quantify loss of PD due to phylogenetically clustered extinction risks on model trees. We build upon Heard and Mooers [22] to incorporate the effects of a larger range of phylogenetic signal in extinction risk, different species' mean extinction risk, tree size, tree balance and distribution of nodes across a phylogeny. In addition, we also utilize the probabilistic measure of future PD, i.e. expected future PD, in our model. Even though our model does not entirely capture the exact shape of the current distribution of extinction risks [13], it does provide a reasonable estimate of the loss of PD, as well as a suitable foundation to investigate our question with other models. Overall, we ask under which conditions and to what extent phylogenetically clustered extinction risks might be necessary and sufficient for the observed pronounced loss of PD projected for the Tree of Life under the current extinction regime.

## Methods

### Generation of model trees

Yule trees [23]–[27] have long served as a null model for various macro-evolutionary phylogenetic studies [28]–[31]. They are less balanced than random tree shapes, though more balanced than many inferred trees [32], [33], and their distribution of edge lengths falls between that expected under adaptive radiations [34] and long-term equilibrial conditions [35]. We simulated two sets of 1000 Yule trees, (speciation rate  = 0.5), one with 64 and one with 128 tips using the appropriate simple-sample approach [SSA; 36]. We checked for the validity of these Yule trees with their gamma statistic [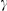, 5]. As expected, we obtained a standard normal distribution of the gamma statistic (unpublished data). We also produced 1000 64-tip completely balanced and unbalanced trees, each with uniformly-distributed internal branch lengths, as well as a set of 1000 64-tip coalescent trees. We set all trees to a common depth value (depth = 1) to facilitate comparisons across simulations.

### Trait simulation and the relevance of excessive phylogenetic signal

We then simulated a set of continuous traits along each tree under the Brownian Motion (BM) model of change, another common model for continuous trait evolution [37], [38]. Specifically, we simulated these traits with different values of Pagel's 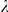 [39] to model different strengths of phylogenetic clustering (further quantified as the relative similarity of tips on a tree compared to expectations from perfect BM) – from 0 (no clustering) to 1 (consistent with perfect Brownian motion, where the expected covariance between two nodes is equal to the height of their first common node). With Revell's framework [40], we were also able to incorporate higher values of 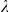 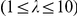 in our data. We categorized the values of lambda that fall under the open interval of 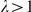 to represent a higher relative similarity of tips compared to expectations from the perfect BM model. This case of elevated values of lambda could be attributed either to mistakes in tree inference, with internal branches being biased short (producing negative gamma values; [41], [42]) or to evolutionary models such as the “Early Burst” (EB) model [43]–[48]. Under this process model, species' morphological trait values evolve more rapidly near the root than expected under the perfect BM model of evolution, followed by a relative stasis towards the tips. This phenomenon causes the trait values at the tips to be relatively more similar within a subclade than expected under perfect BM, producing higher tip disparity (higher variance among, rather than, within subclades) near the root and decreasing tip disparity as the trait approached the tips of a tree [47], [48]. Figure S1 shows disparity through time plots of trait values on a model tree at various values of Pagel's 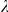 to illustrate this phenomenon. In addition, the Figure S2 represents the evolution of a continuous trait with different phylogenetic signals, quantified by two independent measures of phylogenetic signal (Pagel's λ and Blomberg's K statistic) [39], [46].

### Extinction probabilities and expected future PD

**Figure 2:**
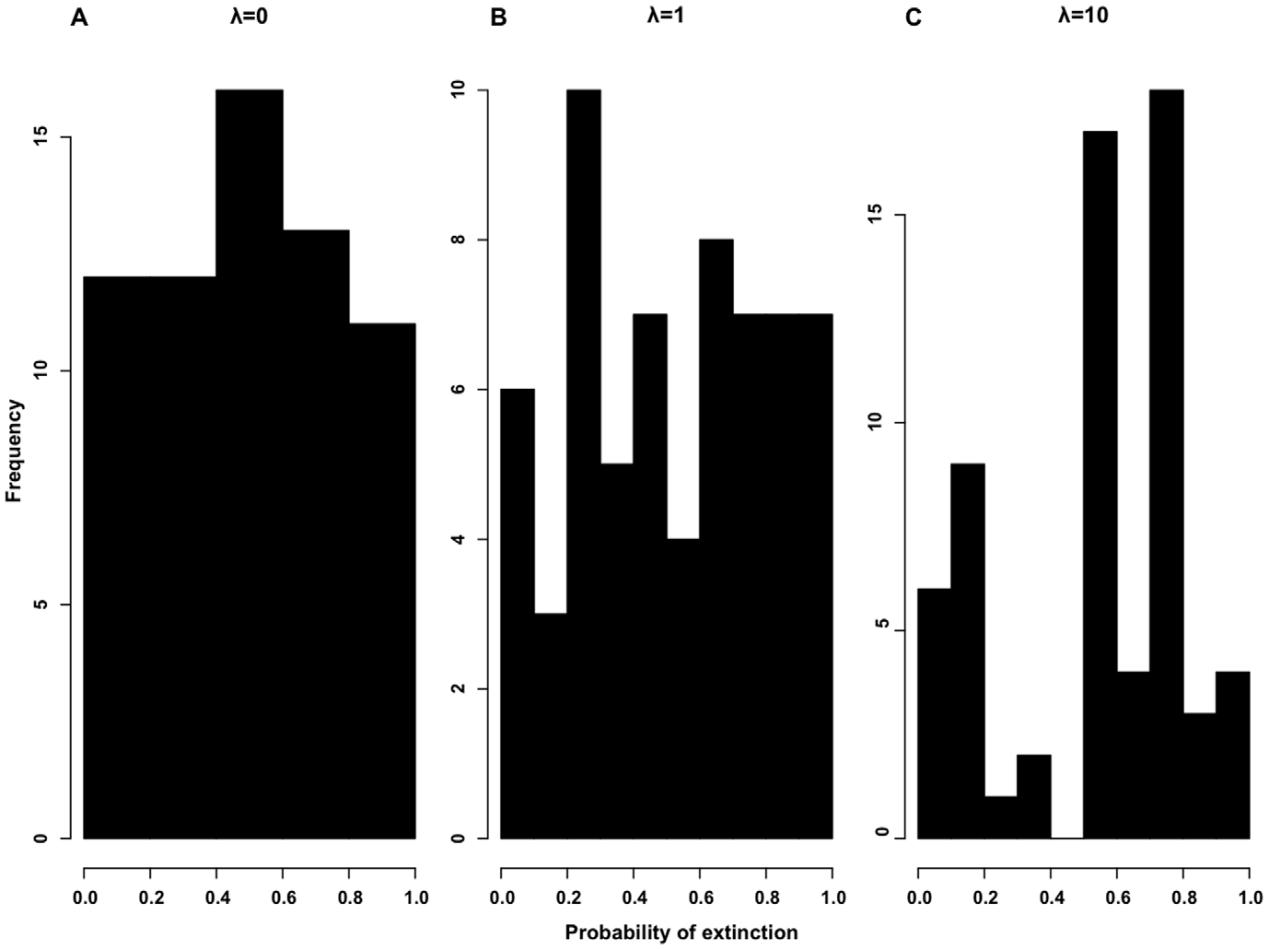
Differences in the extinction risk distributions. Extinction risk distributions across the tips of an example 64-tip Yule tree, modeled for three levels of phylogenetic clustering: 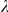 = 0, 1, and 10. Each set has mean p(ext)  = 0.5.

Above, we produced continuous traits on our trees with a range of phylogenetic clustering from 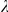  = 0 to 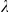  = 10. Next, we transformed the trait values to produce distributions for the p(ext) of the corresponding tips at different levels of phylogenetic clustering using Equation 1 and 2 below. These p(ext) distributions (with a mean value of 0.5, see Figure 2) were then used to calculate the expected future PD of a phylogenetic tree using Equation 3.

For a given single tree, we used the following two transformations: 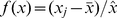
(1)

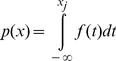
(2)where the term 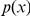 represents the distribution of probability of extinction, 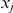 represents the raw final trait value of a particular taxa *j*, while 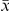 and 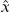 represent the mean and standard deviation of these corresponding traits, respectively. We used three different distributions with a low (0.25), medium (0.50) and high (0.75) mean values of species extinction risk, to better represent the range of mean anthropogenic impacts on p(ext). To simulate these scenarios, we used the original p(ext) distribution (with mean value of 0.5) and transformed (using simple division and/or multiplication) their values to produce other distributions with mean values of 0.25 and 0.75, respectively. We then assigned the extinction vulnerabilities to the corresponding taxa and calculated the expected future PD [E(PD)] of each tree under the “generalized field of bullets” model or the g-FOB model [2]:
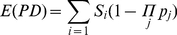
(3)The term 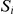 represents the length of an edge *i*, while 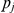 represents the probability of extinction for the corresponding subtended *j*th taxa in a reference phylogeny.

In order to answer our main question, we compared E(PD) on the modeled p(ext) values with E(PD) obtained from the identical tree with shuffled p(ext) values, representing a useful extension of the uniform p(ext) FOB model reported by Nee and May [3] (see also [2]). We report the results [E(PD) and p(ext) distribution] for each set of 1000 trees, at different strengths of phylogenetic clustering and mean extinction risk. All tree simulations and data analysis were carried in the R programming environment [49], with the primary help of the “Ape” [50] and “Geiger” [51] packages.

## Results

**Figure 3:**
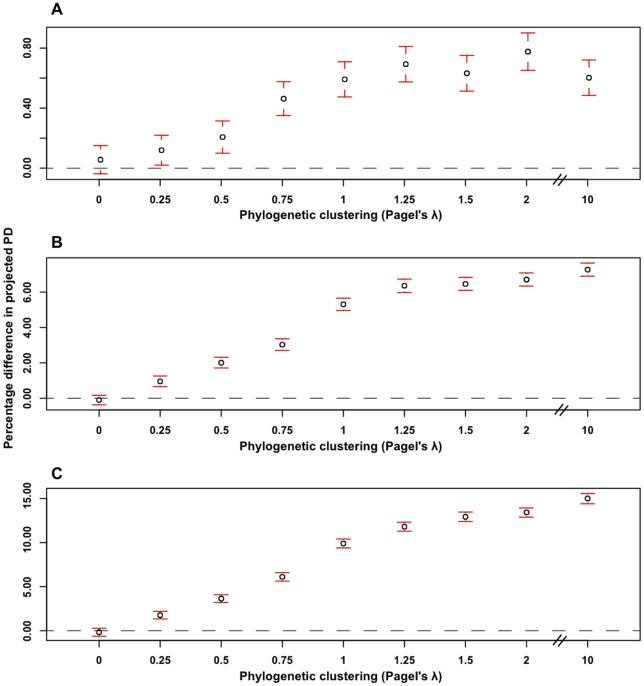
Percentage difference in projected PD with phylogenetic clustering in 64-tip Yule trees. **A)** mean p(ext)  = 0.25, **B)** mean p(ext)  = 0.5, and **C)** mean p(ext)  = 0.75. Data points in percentage denote the amount of additional loss of projected PD (relative to random extinction) with increasing phylogenetic clustering. Dashed line indicates that loss under random extinction. Error bars around points represent the 95% confidence interval with a sample size of 1000 trees. Note differences in vertical axes.

We observed no noticeable effect in any of our results with an increase in the size of Yule trees except a minor decrease in the confidence limits on our estimates (compare Figure 3 and Figure S3). We therefore only reported results for 64 tips below.

### Extinction risk distributions

Varying the mean extinction risk and lambda produced different distributions of p(ext) across the tips of our trees. At no phylogenetic signal 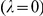, we observed a relatively uniform distribution of p(ext) following our transformation, equivalent to a case of fully random extinction (Figure 2A). At 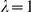, we observed a quasi-normal distribution (Figure 2B). At higher phylogenetic signal, risk values diverged away from the mean value, resulting in a bimodal distribution at the highest lambda value (Figure 2C).

### Mean extinction risk, and the relationship between Pagel's 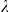 and the loss of PD

We defined the term *Percentage difference in projected PD* or*%ΔE(PD)* as:

%ΔE(PD)
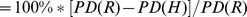
(4)

where 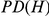 represented the projected or expected future PD calculated under a certain level of heritable extinction risk, while 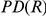 represented that quantity under a randomized set of those identical extinction risks.

Throughout our test scenarios, we observed consistent patterns in the relationship between the amount of phylogenetic clustering and the percentage difference in projected PD; we presented these patterns for 64-tip Yule trees in Figure 3. Overall, and as expected, when there was no phylogenetic clustering 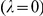,%ΔE(PD) was centered on zero. We observed a minimal increase in%ΔE(PD) with an increase in Pagel's 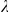. In addition, the rate of increase in%ΔE(PD) reached relative stasis as we increased the phylogenetic signal beyond the perfect Brownian Motion model (Figure 3).

Further, we also observed the same qualitative pattern in%ΔE(PD) as a function of phylogenetic signal across all three scenarios of different mean extinction risk (compare Figure 3A, B, and C). For a particular scenario (size, balance, temporal change in the rate of diversification, etc.), we observed the maximum value of%ΔE(PD) to be consistently associated with the highest mean extinction risk. Though remaining absolutely low, the maximum value of%ΔE(PD) increased many-fold with a doubling of mean extinction risk. For instance, 64-tip Yule trees produced a maximum loss of ∼0.7% at mean p(ext)  = 0.25, and a maximum loss of ∼7%, at mean p(ext) = 0.50 (compare Figure 3A and 3B). Importantly, across all cases, the values of%ΔE(PD) were not significantly different at phylogenetic signals equal to and exceeding the perfect Brownian Motion model for low mean extinction threat (Figure 3A).

### Effect of tree topology on loss of PD

On perfectly balanced trees (with Yule edge-lengths), the distribution of%ΔE(PD) as a function of phylogenetic signal was similar to the one produced with randomly generated Yule trees (with 64 or 128 tips), with a maximum value of ∼14% at our highest chosen mean extinction risk (Figure S4). However, in the case of unbalanced trees (with Yule topologies), we observed a *decline* in the values of%ΔE(PD) with increasing phylogenetic signal (Figure S5). This led to slight negative mean values of%ΔE(PD).

### An extreme case of 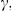 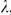 and 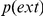


We also tested our question on the extreme model of diversification. The extreme case, i.e., coalescent trees, produced the highest (among all of our tested scenarios) values of%ΔE(PD) as a function of phylogenetic signal (Figure S6). Under this case, the largest amount of loss was relatively substantial (∼20%), but occurred only in the presence of both the highest mean extinction risk as well as extremely excessive phylogenetic signal (Figure S6C).

## Discussion

We highlight two main findings. We interpret our results to mean that phylogenetically clustered extinction risks alone are not sufficient to explain appreciable extra losses of E(PD) [%ΔE(PD)] due to projected extinction. In addition, the overall amount of this additional loss does not substantially scale with tree size, tree balance and extreme cases of temporal changes in the rates of diversification, respectively, though mean p(ext) has a strong relative effect. Rather, we require at least a combination of these factors with uncommonly excessive values to drive a substantial loss of PD.

Nee and May [3] previously suggested that random pruning events led to a minimal loss of PD under a uniform p(ext) distribution. As well as not accounting for differences in the species level of endangerment [7], [13], [52], [53] their modeled trees only made use of the extreme topology, i.e., the coalescent model. In contrast, our results are based on a generalized version [2] of Nee and May's [3] model and our modeled Yule trees are much closer in line with the shapes of inferred trees [54]. Finally, we investigated several aspects of phylogenies that could influence the percentage difference in projected PD. Results suggest that the introduction of phylogenetically patterned extinction risks alone leads to a low loss of the percentage difference in projected PD (Figure 3).

### The general pattern

Under random extinction scenarios such as those modeled by Nee and May [3], species' extinction risks are allocated independent of their location in the corresponding phylogeny. This scenario does not link the extinction risk of a species with its biology (and thus its evolutionary history). As predicted, we found no effect on%ΔE(PD) at very low levels of phylogenetic clustering (Figure 3). When traits displayed relatively more phylogenetic signal (related taxa show similar trait values) non-random extinction is expected [7] and observed (our results).

However, contrary to previous studies [7], [11], [21], non-randomness (phylogenetic clustering) in species' extinction risk alone does not contribute towards a substantial loss of PD, even if this non-random effect exceeds the perfect Brownian Motion model on Yule topologies (Figure 3). Indeed, higher than 'expected' levels of phylogenetic clustering (i.e., Pagel's 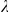 >>1) causes a decelerating rate for the additional loss of PD. As described earlier, at higher phylogenetic signals, simulated extinction risks deviate away from their mean value relative to cases of lower clustering (Figure 2). Since the probability function has a restricted domain 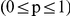, extinction probabilities cannot diverge away from their mean value without bound and so the increase in the percentage difference in projected PD is limited. At higher phylogenetic signals, this slows the rate of increase in this extra loss. Of course, more sophisticated models of extinction threat might lead to different outcomes.

### Quantifying the effects of mean extinction risk on Yule trees

The extra loss of PD [%ΔE(PD)] also changes with different values of mean extinction risk (Equation 4). Logically, an increase in mean extinction risk will decrease both the projected PD due to random extinction 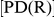, and projected PD due to phylogenetically clustered extinction 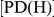 of similar magnitude, producing a small effect on the numerator of%ΔE(PD) (Equation 4). However, the denominator 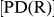 decreases directly with increasing mean p(ext). Therefore, we expect%ΔE(PD) to increase with increase in mean extinction risk. Consistent with this, we observed the largest value of%ΔE(PD) at cases of highest mean threat to species survival (mean p(ext)  = 0.75). However, this maximal (but still low) percentage difference in projected PD does not scale linearly with the mean extinction risk. Again, this is due to the fact that we must limit range of extinction risk (p(ext) cannot be >1) and simultaneously maintain a constant mean value. This leads to a slight decrease in the variance of the distribution of extinction risk as mean p(ext) goes up, which potentially decreased the difference between the measures of 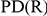 and 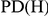. This is a limitation of our model, and we suggest future models should minimize differences in the variance of probability functions while maintaining various constant mean values.

Our results also indicate that there is a non-significant increase in the percentage difference in projected PD beyond the perfect Brownian Motion model of change at low mean extinction risk (mean p(ext) = 0.25; see Figure 3A). This is expected because the combination of low mean extinction risk and a deceleration in the rate of increase in%ΔE(PD) at higher phylogenetic signals causes for an irrelevant additional loss of PD. Furthermore, the percentage difference in projected PD is also not much different (<0.6%) at other levels of phylogenetic clustering.

### Role of tree balance in the loss of PD

We find an interesting pattern in the percentage difference in projected PD on maximally unbalanced trees. Overall, we see minimal loss. If mean p(ext) is high we actually lose more PD under random than under clustered extinction. Our unbalanced trees had very short internal edges, and a few very long pendant edges. Thus, when risks are clustered on an unbalanced tree, taxa with longer pendant edges are less likely to be pruned than those with shorter edges, decreasing the percentage difference in projected PD.

Alternatively, a balanced tree of the same size produces a loss similar to a randomly generated Yule tree. Here, every edge is of the same length and there is the maximum amount of topological redundancy. We therefore expect to lose more PD from lineages that share similar extinction risks.

### The extreme case of diversification

We find that the amount of PD lost on coalescent trees is the most sensitive to particular values of our chosen parameters. For instance, we do find a similar quantitative pattern to Yule trees in the percentage difference in projected PD in coalescent trees at very low and moderate levels of 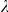 and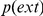. However, on coalescent trees at high 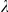 and p(ext), we can observe substantial loss of PD (∼20%). This scenario, in addition to being extreme, is only relevant if real inferred trees are well represented by the coalescent model. Recent studies [55], [56] indicate that this is unlikely.

In our study, we were unable to incorporate the exact shape of the distribution of extinction probabilities suggested by recent assessments [13]. This distribution is heavily skewed, assigning the majority of the species with low threat and a few species with high vulnerability. Future studies could incorporate this distribution in their model to get a much better picture of the loss of PD from the Tree of Life.

To conclude, we propose that other non-random processes in addition to phylogenetic clustering of species' extinction risks must explain the appreciable loss of PD projected on real trees. Inferred trees do have very isolated small clades, and the highest extinction risk may be found in such small isolated clades [12], [19], [35], [54]. Why this is the case is an open and fairly urgent question.

## Supporting Information

Figure S1 **Disparity through time (DTT) plots for trait values at various phylogenetic signals.** The x-axis indicates the relative time elapsed or age of clade with 0 representing its origin and 1 representing its current age. Solid line indicates the observed disparity values, whereas the dashed line represents the mean of 100 simulated disparity values expected under the Brownian Motion model. **A)** through **G)** represent various DTT plots at increasing phylogenetic signal simulated on one example tree. High relative disparity is indicative of more variation in trait values within subclades than between subclades, which are found near the tips of a phylogeny with the trait displaying no phylogenetic signal. In contrast, low relative disparity is indicative of less variation in trait values within subclades than between subclades, which are found near the tips of a phylogeny with the trait displaying an excessive phylogenetic signal.
(TIFF)

Figure S2
**Traitgrams and the two measures of phylogenetic signal.** The three traitgrams show the evolution of a continuous trait evolving under different phylogenetic signals. The x-axis represents a continuous scale of species' trait values and node depths represent the phylogenetic edge lengths. Traitgram in **A)** represents the evolution of a trait with no phylogenetic signal (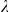 = 0), while traitgrams in **B)** and **C)** represent the evolution of a trait under the perfect BM model and when evolution exceeds the perfect BM model (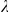 = 1 and 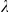  =  10), respectively. Each case of evolution is represented by two independent measures of phylogenetic signal (Pagel's λ and Blomberg's K statistic).(TIFF)

Figure S3 **Quantifying percentage difference in projected PD with phylogenetic clustering in 128-tip Yule trees. A)** mean p(ext)  = 0.25, **B)** mean p(ext)  = 0.5, and **C)** mean p(ext)  = 0.75. Data points in percentage denote the amount of additional loss of projected PD (relative to random extinction) with increasing phylogenetic clustering. Dashed line indicates that loss under random extinction. Error bars around points represent the 95% confidence interval with a sample size of 1000 trees. Note differences in vertical axes.
(TIFF)

Figure S4 **The additional loss of PD as function of phylogenetic clustering in 64-tip balanced Yule trees. A)** mean p(ext)  = 0.25, **B)** mean p(ext)  = 0.5, and **C)** mean p(ext)  = 0.75. Data points in percentage denote the amount of additional loss of projected PD (relative to random extinction) with increasing phylogenetic clustering. Dashed line indicates that loss under random extinction. Error bars around points represent the 95% confidence interval with a sample size of 1000 trees. Note differences in vertical axes.
(TIFF)

Figure S5 **The additional loss of PD as function of phylogenetic clustering in 64-tip unbalanced Yule trees. A)** mean p(ext)  = 0.25, **B)** mean p(ext)  = 0.5, and **C)** mean p(ext)  = 0.75. Data points in percentage denote the amount of additional loss of projected PD (relative to random extinction) with increasing phylogenetic clustering. Dashed line indicates that loss under random extinction. Error bars around points represent the 95% confidence interval with a sample size of 1000 trees. Note differences in vertical axes.
(TIFF)

Figure S6 **Percentage difference in projected PD as a function of phylogenetic clustering in 64-tip coalescent trees. A)** mean p(ext)  = 0.25, **B)** mean p(ext)  = 0.5, and **C)** mean p(ext)  = 0.75. Data points in percentage denote the amount of additional loss of projected PD (relative to random extinction) with increasing phylogenetic clustering. Dashed line indicates that loss under random extinction. Error bars around points represent the 95% confidence interval with a sample size of 1000 trees. Note differences in vertical axes.
(TIFF)
