# Supplementary figures and images for: Phylogenetically Clustered Extinction Risks Do Not Substantially Prune the Tree of Life
Source: PLoS One. 2011 Aug 10;6(8):e23528. doi: 10.1371/journal.pone.0023528 (PMC3154466; doi:10.1371/journal.pone.0023528)

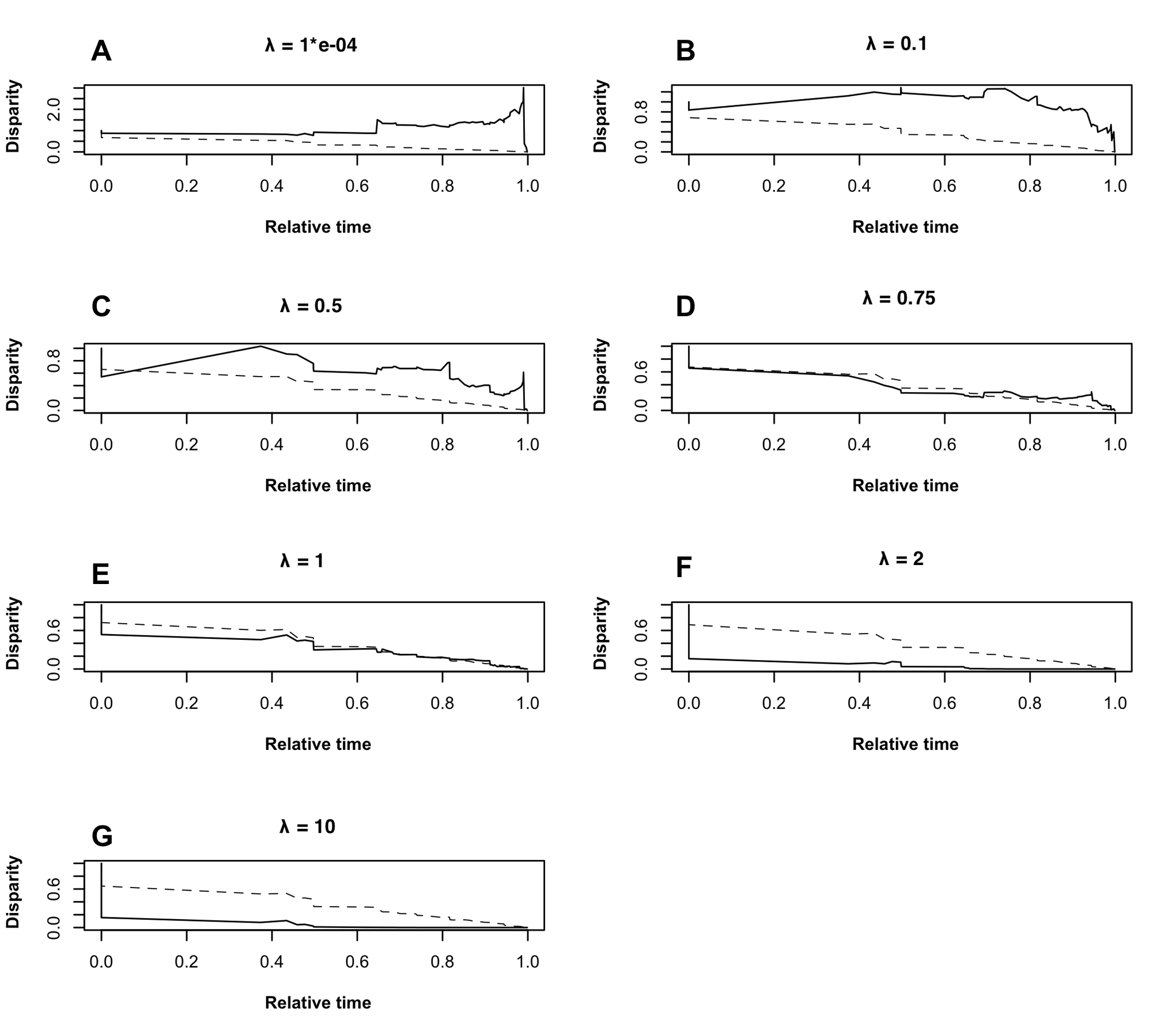

Supplement: Figure S1 — Disparity through time (DTT) plots for trait values at various phylogenetic signals. The x-axis indicates the relative time elapsed or age of clade with 0 representing its origin and 1 representing its current age. Solid line indicates the observed disparity values, whereas the dashed line represents the mean of 100 simulated disparity values expected under the Brownian Motion model. A) through G) represent various DTT plots at increasing phylogenetic signal simulated on one example tree. High relative disparity is indicative of more variation in trait values within subclades than between subclades, which are found near the tips of a phylogeny with the trait displaying no phylogenetic signal. In contrast, low relative disparity is indicative of less variation in trait values within subclades than between subclades, which are found near the tips of a phylogeny with the trait displaying an excessive phylogenetic signal. (TIFF) [file pone.0023528.s001.tiff]

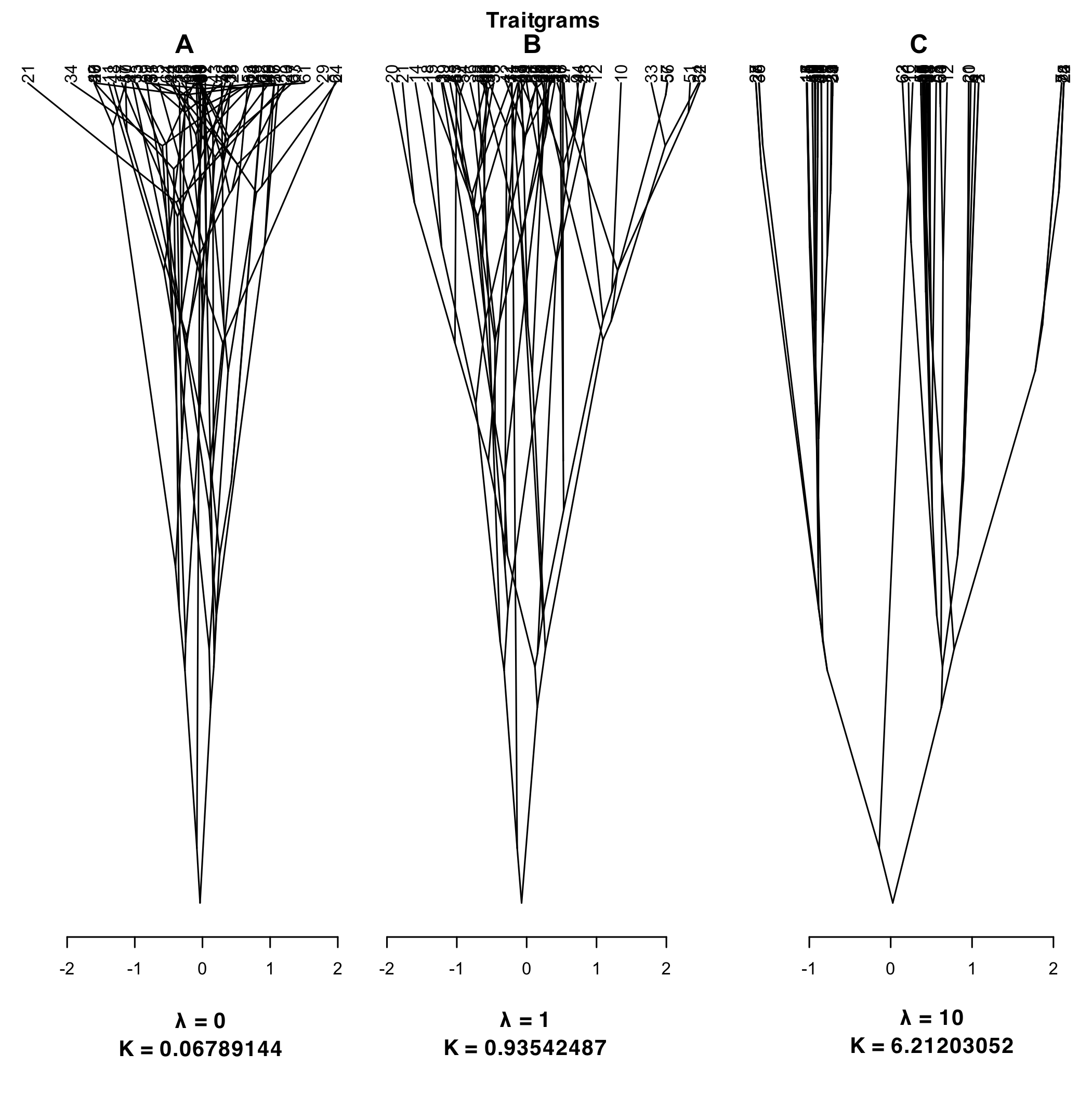

Supplement: Figure S2 — Traitgrams and the two measures of phylogenetic signal. The three traitgrams show the evolution of a continuous trait evolving under different phylogenetic signals. The x-axis represents a continuous scale of species' trait values and node depths represent the phylogenetic edge lengths. Traitgram in A) represents the evolution of a trait with no phylogenetic signal ( = 0), while traitgrams in B) and C) represent the evolution of a trait under the perfect BM model and when evolution exceeds the perfect BM model ( = 1 and = 10), respectively. Each case of evolution is represented by two independent measures of phylogenetic signal (Pagel's λ and Blomberg's K statistic). (TIFF) [file pone.0023528.s002.tiff]

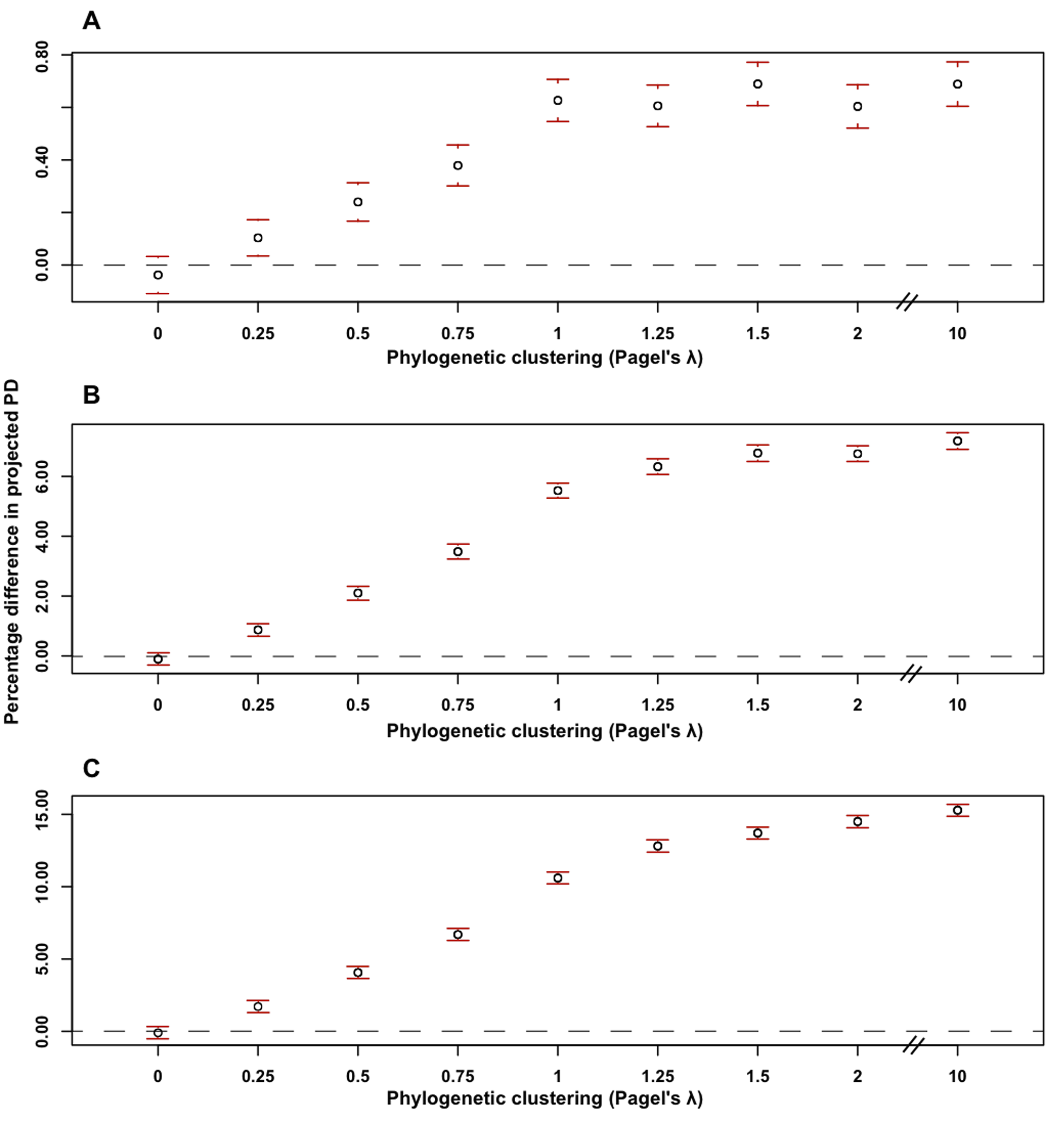

Supplement: Figure S3 — Quantifying percentage difference in projected PD with phylogenetic clustering in 128-tip Yule trees. A) mean p(ext) = 0.25, B) mean p(ext) = 0.5, and C) mean p(ext) = 0.75. Data points in percentage denote the amount of additional loss of projected PD (relative to random extinction) with increasing phylogenetic clustering. Dashed line indicates that loss under random extinction. Error bars around points represent the 95% confidence interval with a sample size of 1000 trees. Note differences in vertical axes. (TIFF) [file pone.0023528.s003.tiff]

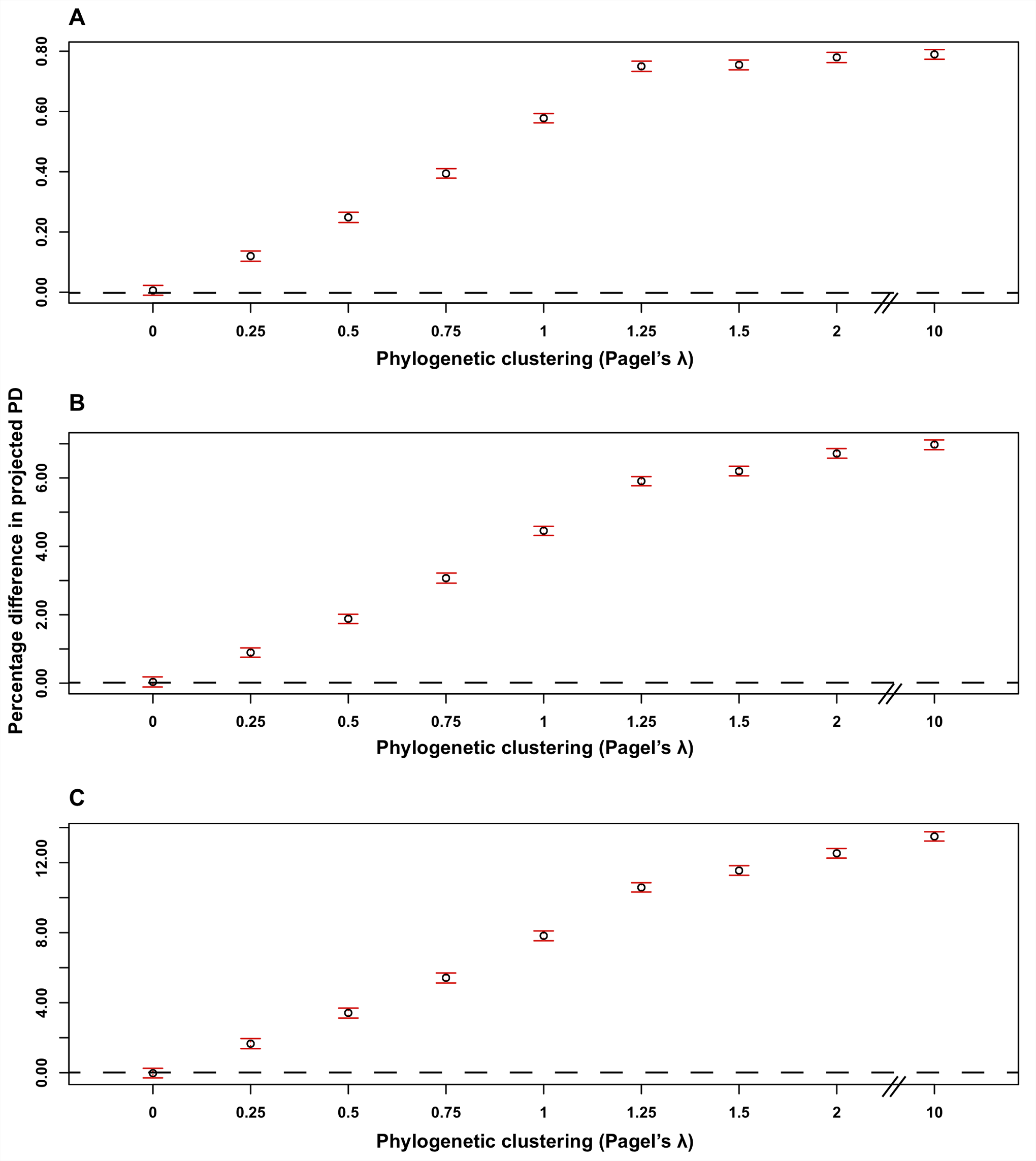

Supplement: Figure S4 — The additional loss of PD as function of phylogenetic clustering in 64-tip balanced Yule trees. A) mean p(ext) = 0.25, B) mean p(ext) = 0.5, and C) mean p(ext) = 0.75. Data points in percentage denote the amount of additional loss of projected PD (relative to random extinction) with increasing phylogenetic clustering. Dashed line indicates that loss under random extinction. Error bars around points represent the 95% confidence interval with a sample size of 1000 trees. Note differences in vertical axes. (TIFF) [file pone.0023528.s004.tiff]

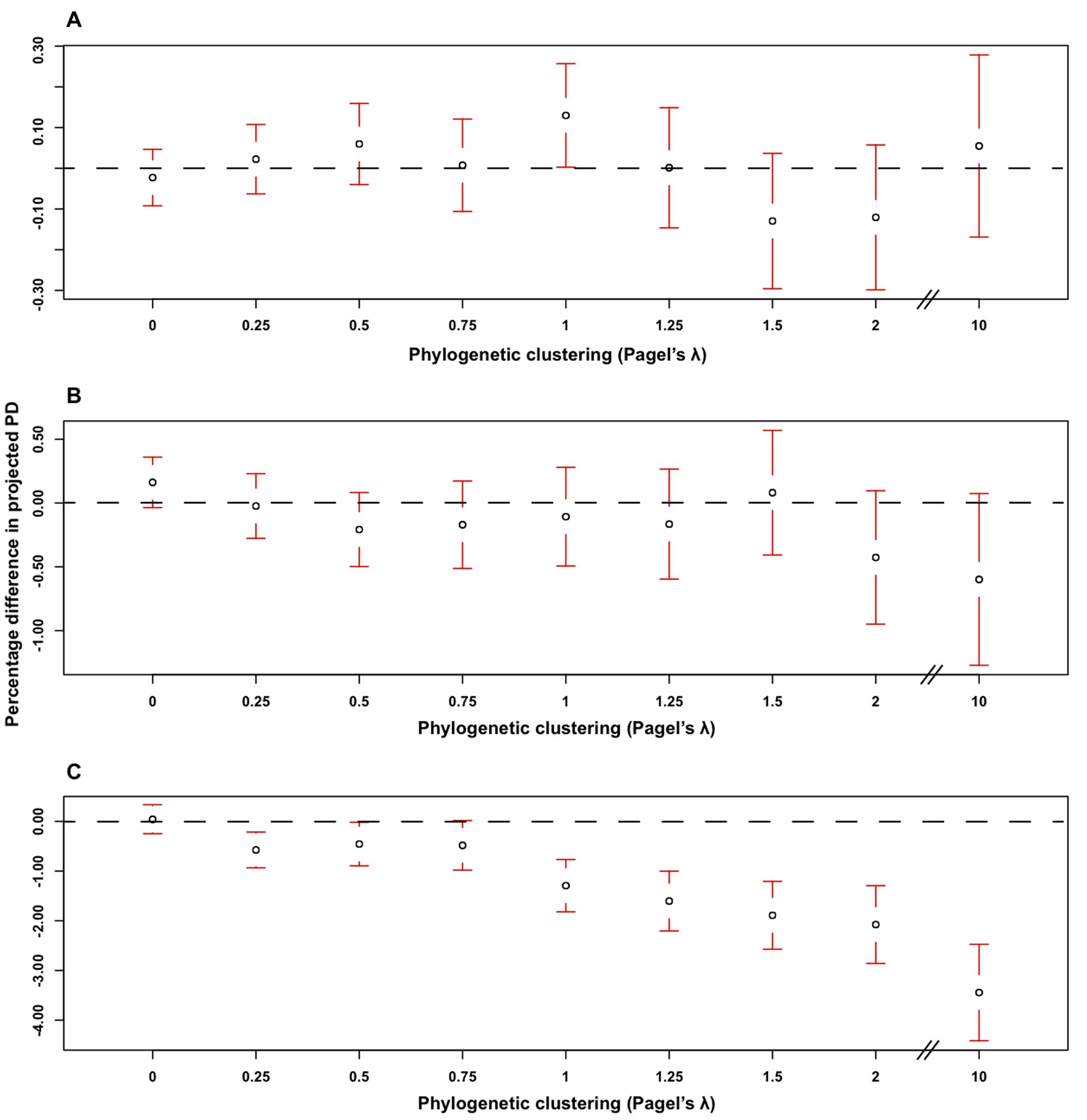

Supplement: Figure S5 — The additional loss of PD as function of phylogenetic clustering in 64-tip unbalanced Yule trees. A) mean p(ext) = 0.25, B) mean p(ext) = 0.5, and C) mean p(ext) = 0.75. Data points in percentage denote the amount of additional loss of projected PD (relative to random extinction) with increasing phylogenetic clustering. Dashed line indicates that loss under random extinction. Error bars around points represent the 95% confidence interval with a sample size of 1000 trees. Note differences in vertical axes. (TIFF) [file pone.0023528.s005.tiff]

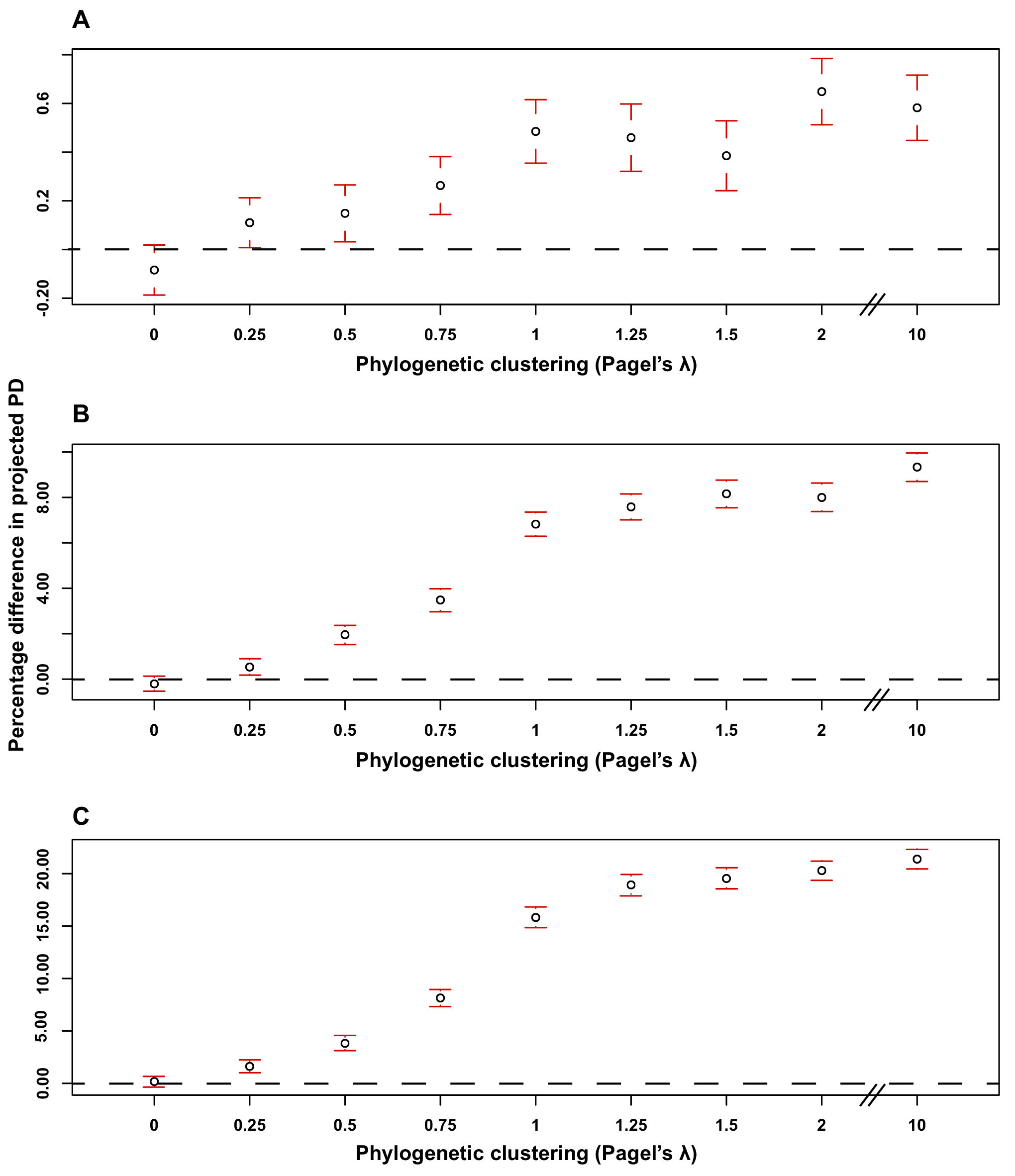

Supplement: Figure S6 — Percentage difference in projected PD as a function of phylogenetic clustering in 64-tip coalescent trees. A) mean p(ext) = 0.25, B) mean p(ext) = 0.5, and C) mean p(ext) = 0.75. Data points in percentage denote the amount of additional loss of projected PD (relative to random extinction) with increasing phylogenetic clustering. Dashed line indicates that loss under random extinction. Error bars around points represent the 95% confidence interval with a sample size of 1000 trees. Note differences in vertical axes. (TIFF) [file pone.0023528.s006.tiff]
